# Rare Combination of Multiple Endocrine Neoplasia 1 with Medullary Thyroid Carcinoma and Clinical Value of ^68^ Ga-DOTATATE PET/CT in Diagnosing Different Lesions and Exploring Theranostic Strategy

**DOI:** 10.1055/s-0044-1791818

**Published:** 2024-10-25

**Authors:** Parth Baberwal, Rahul V. Parghane, Sandip Basu

**Affiliations:** 1 Radiation Medicine Centre, Bhabha Atomic Research Centre, Tata Memorial Hospital Annexe, Mumbai, Maharashtra, India; 2 Homi Bhabha National Institute, Mumbai, Maharashtra, India

**Keywords:** multiple endocrine neoplasia type 1 syndrome, parathyroid carcinoma, medullary thyroid carcinoma, ^68^ Ga-DOTATATE PET/CT, neuroendocrine tumor, theranostics

## Abstract

Multiple endocrine neoplasia type 1 (MEN1) syndrome is characterized by the presence of hyperplastic or neoplastic tumors in the parathyroid, pituitary, and gastroenteropancreatic endocrine tissues. The presence of lesions in at least two out of the three glands (pituitary, parathyroid, and pancreas) is indicative of MEN1 syndrome. Medullary thyroid carcinoma (MTC) is a type of tumor that originates from the parafollicular C cells of the thyroid gland. It is frequently found as part of MEN2 syndrome. MEN1 with MTC is a relatively uncommon occurrence. We report a rare case of MTC that later on developed parathyroid carcinoma, pituitary microadenoma, pancreatic neuroendocrine tumor (NET), and duodenal NET. The case was identified as part of MEN1 syndrome using exome sequencing and somatostatin receptor–based functional imaging ^68^ Ga-DOTATATE positron emission tomography/computed tomography was employed for exploring theranostic strategy in the management of the patient.

## Introduction

Multiple endocrine neoplasia type 1 (MEN1) syndrome is a hereditary endocrine neoplasm syndrome that generally consists of tumors from the anterior pituitary gland, parathyroid gland, and gastroenteropancreatic tract neuroendocrine tumors (NETs). 1 The occurrence of medullary thyroid carcinoma (MTC) in a patient with MEN1 syndrome is extremely uncommon. We present a rare case of MTC that later developed parathyroid carcinoma, pituitary microadenoma, and pancreatic and duodenal NET, as part of MEN1 syndrome.

## Case Report

**Fig. 1:**
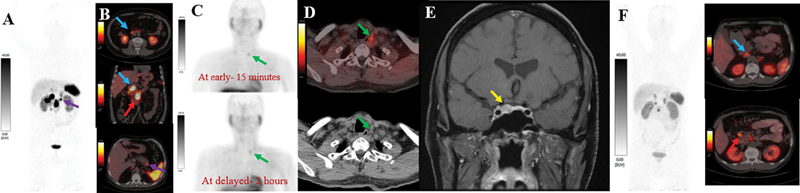
^68^ Ga-DOTATATE PET/CT scan with ( **A** ) maximum intensity projection image and ( **B** ) fused axial and coronal images showed somatostatin receptor (SSTR) expressing duodenal neuroendocrine tumor (NET; *blue arrows* ), pancreatic NET ( *purple arrow* ), and metastatic mesenteric lymph node ( *red arrow* ). ( **C** ) ^99m^ Tc-MIBI scan with planar images at early-15 minutes and delayed-2 hours show tracer retention in the left inferior thyroid region. ( **D** ) Single photon emission computed tomography (SPECT)/computed tomography (CT) of ^99m^ Tc-MIBI scan with fused axial and axial CT images showed tracer localization to subcentimeter-sized soft-tissue lesion in left inferior paratracheal region ( *green arrow* ) indicating the parathyroid tumor. ( **E** ) Magnetic resonance imaging (MRI) with coronal T1 postcontrast image showing 0.5 × 0.7 cm lesion in the right half of pituitary gland associated with hypoenhancement pattern suggestive of microadenoma ( *yellow arrow* ). The patient received four cycles of ^177^ Lu-DOTATATE peptide receptor radionuclide therapy (PRRT). ( **F** ) Post-PRRT, ^68^ Ga-DOTATATE PET/CT scan showed reduction in size and SSTR uptake of duodenal and mesenteric NET lesions as compared with baseline ^68^ Ga-DOTATATE PET/CT scan ( **A** and **B** ), suggestive of a favorable response to PRRT.

A 36-year-old male patient initially complained of neck swelling; later a nodule was found in the right lobe of the thyroid gland, which was finally diagnosed as MTC in 2009. Postexcision of the thyroid nodule, he was asymptomatic with a serum calcitonin level of less than 2.0 pg/mL. But after period of 6 months, the patient again complained of neck swelling. Ultrasonography (USG) of the neck showed a 2.0 × 1.4 cm lesion in the superior pole of the right thyroid lobe. Then the patient underwent total thyroidectomy with neck dissection in 2009, which demonstrated parathyroid carcinoma (mindbomb homolog-1 [MIB-1] labeling index: 4%) infiltrating the surrounding muscle. Immunohistochemistry (IHC) was strongly positive for chromogranin A and negative for insulinoma associated protein, calcitonin, and thyroid transcription factor-1 (TTF-1). Postsurgery (total thyroidectomy with neck dissection), the patient complained of abdominal discomfort in 2022, which was investigated with USG of the abdomen. A hypoechoic nodule of 2.0 × 1.9 cm was found in the body of the pancreas. Subsequently, the patient underwent an upper gastrointestinal endoscopy with guided biopsy, which demonstrated a well-differentiated grade II pancreatic NET (MIB-1 labeling index of 6%) and grade I NET of the duodenum (MIB-1 labeling index of 1%). On IHC, the tumor cells expressed chromogranin A and synaptophysin. The patient underwent ^68^ Ga-DOTATATE PET/CT scan, which showed somatostatin receptor (SSTR) expressing duodenal lesions, pancreatic lesion, and SSTR expressing mesenteric lymph node as shown in Fig. 1A and B . In 2022, the serum parathyroid hormone level was found to be raised (108 pg/mL). Subsequently, the patient underwent [ ^99m^ Tc]Tc-sestamibi scan with single photon emission computed tomography (SPECT)/computed tomography (CT), which showed a tumor in the left inferior parathyroid as shown in Fig. 1C and D . In 2022, the serum prolactin level was found to be raised (480 ng/mL). Magnetic resonance imaging (MRI) of head region showed a hypoenhancement pattern lesion in the right half of the pituitary gland, which was suggestive of microadenoma ( Fig. 1E ) leading to the clinical and imaging diagnosis of MEN1 syndrome in 2022. The patient underwent genetic testing with germline clinical exome testing of 232 genes, which detected pathogenic germline heterogeneous gene mutation in exon 2 of the MEN1 gene (c.249_252del). For parathyroid adenoma and pituitary microadenoma, the patient was managed with medical treatment with tablets. Cinacalcet 30 mg once daily was prescribed for parathyroid adenoma–related hypercalcemia and cabergoline for pituitary microadenoma. The patient received ^177^ Lu-DOTATATE peptide receptor radionuclide therapy (PRRT) for duodenal and pancreatic NET with mesenteric lymph nodal lesions. The patient received four cycles of PRRT from 2022 to 2023 with a total cumulative dose of 800 mCi (200 mCi per cycle at an interval of 8–12 weeks). Post-PRRT, ^68^ Ga-DOTATATE PET/CT scan ( Fig. 1F ) showed a reduction in size and SSTR uptake in duodenal and mesenteric NET lesions as compared with baseline ^68^ Ga-DOTATATE PET/CT scan ( Fig. 1A and B ), suggestive of a favorable response to PRRT. He is alive, doing well, and on regular follow-up at our institute.

## Discussion

MEN1 syndrome (also known as Wermer's syndrome) consists of hyperplastic or neoplastic tumors of the pituitary, parathyroid and gastroenteropancreatic endocrine tissues. 1 The MEN1 gene is located on chromosome 11q13.1. It consists of 10 exons that encode a protein known as menin. Germline mutation itself is not sufficient to cause MEN1 syndrome and loss of unaffected allele is necessary for clinical symptoms to develop. However, 10% of MEN1 syndrome patients do not harbor MEN1 mutations. 2 Lesion detection in at least two out of three glands (pituitary, parathyroid, and gastro-entero-pancreas) is diagnostic for MEN 1 syndrome. MTC is a tumor arising from the parafollicular C cells of the thyroid gland and is more commonly a component of MEN2 syndrome. MEN1 with MTC is a rather rare entity and there have been only four case reports so far. 3 4 5 6 In our case report, the patient had duodenal and pancreatic NET, prolactinoma, and primary hyperparathyroidism secondary to parathyroid tumor. Another very interesting finding in this case was the incidence of parathyroid carcinoma as a part of MEN1 syndrome. The most common manifestation of MEN1 syndrome is primary hyperparathyroidism. 7 Parathyroid carcinoma accounts for less than 1% of cases of primary hyperparathyroidism. According to the literature, there have been only 20 documented cases of parathyroid cancer in persons with MEN1 syndrome. 8

To summarize, MEN1 syndrome involves the parathyroid, pancreatic, and pituitary glands. To identify abnormalities in these glands and diagnose MEN1 syndrome early, various imaging methods are necessary. Functional imaging methods such as ^68^ Ga-DOTATATE PET/CT scans not only identify lesions but also offer the potential for theranostics and treatment of NET in MEN1 syndrome.

## Conclusion

Rarely, MTC may occur in MEN1 syndrome along with parathyroid carcinoma, pituitary prolactinoma, and duodenal and pancreatic NET, necessitating the use of various imaging modalities to identify the involvement of such an atypical case along with lesions in different organs in MEN1 syndrome. The utilization of ^68^ Ga-DOTATATE PET/CT imaging enables the identification of multiple lesions including gastroenteropancreatic NET and offers a promising theranostic strategy for individuals with MEN1 syndrome.
